# Building a virtual summer research experience in cancer for high school and early undergraduate students: lessons from the COVID-19 pandemic

**DOI:** 10.1186/s12909-021-02861-y

**Published:** 2021-08-09

**Authors:** Timothy W. Corson, Shannon M. Hawkins, Elmer Sanders, Jessica Byram, Leigh-Ann Cruz, Jacob Olson, Emily Speidell, Rose Schnabel, Adhitya Balaji, Osas Ogbeide, Julie Dinh, Amy Hinshaw, Laura Cummings, Vicki Bonds, Harikrishna Nakshatri

**Affiliations:** 1 Indiana University Melvin and Bren Simon Comprehensive Cancer Center, Indiana University, Indianapolis, IN 46202 USA; 2 Department of Ophthalmology, Indiana University School of Medicine, Indianapolis, IN 46202 USA; 3 Department of Biochemistry and Molecular Biology, Indiana University School of Medicine, Indianapolis, IN 46202 USA; 4 Department of Obstetrics and Gynecology, Indiana University School of Medicine, Indianapolis, IN 46202 USA; 5 Indiana Clinical and Translational Sciences Institute, Indianapolis, IN 46202 USA; 6 K-12 STEM Program, Indiana University School of Medicine, Indianapolis, IN 46202 USA; 7 Department of Anatomy, Cell Biology and Physiology, Indiana University School of Medicine, Indianapolis, IN 46202 USA; 8 Riverside High School, Indianapolis, IN 46208 USA; 9 Decatur Central High School, Indianapolis, IN 46221 USA; 10 Indiana University, Bloomington, IN 47405 USA; 11 Lawrence Township Schools, Indianapolis, IN 46226 USA; 12 Herron High School, Indianapolis, IN 46202 USA; 13 Pipeline and Pre-Doctoral Programs, Indiana University School of Medicine, Indianapolis, IN 46202 USA; 14 Department of Surgery, Indiana University School of Medicine, C218C, 980 West Walnut Street, Indianapolis, IN 46202 USA; 15 VA Roudebush Medical Center, Indianapolis, IN 46202 USA

**Keywords:** Research education, Mentoring, Virtual education, Pipeline program, Curriculum development

## Abstract

**Background:**

The COVID-19 pandemic posed a unique challenge for summer research programs in 2020, particularly for programs aimed at hands-on experience for younger trainees. The Indiana University Melvin and Bren Simon Comprehensive Cancer Center supports two pipeline programs, which traditionally immerse high school juniors, seniors, and early undergraduate students from underrepresented populations in science in hands-on projects in cancer biology labs. However, due to social distancing policies during the pandemic and reduction of research operations, these students were not physically allowed on campus. Thus, the authors set out to strategically pivot to a wholly virtual curriculum and evaluate the Virtual Summer Research Experience in Cancer outcomes.

**Methods:**

The virtual program included four components: 1. a core science and professional development curriculum led by high school teachers and senior undergraduates; 2. faculty-delivered didactic sessions on cancer science; 3. mentored, virtual research projects with research faculty; and 4. online networking events to encourage vertical mentoring. Outcomes data were measured using a locally created 11-item Research Preparation Scale, daily electronic feedback, and weekly structured evaluation and feedback via Zoom.

**Results:**

Outcome data suggested high self-reported satisfaction with the virtual program. Outcome data also revealed the importance of coordination between multiple entities for seamless program implementation. This includes the active recruitment and participation of high school teachers and further investment in information technology capabilities of institutions.

**Conclusions:**

Findings reveal a path to educate and train high school and early undergraduate students in cancer research when hands-on, in-person training is not feasible. Virtual research experiences are not only useful to engage students during public health crises but can provide an avenue for cancer centers to expand their cancer education footprints to remotely located schools and universities with limited resources to provide such experiences to their students.

**Supplementary Information:**

The online version contains supplementary material available at 10.1186/s12909-021-02861-y.

## Background

The COVID-19 pandemic resulted in significant challenges to the United States healthcare system [1, 2]. Many Academic Health Centers and Cancer Centers were charged with maintaining the traditional tripartite mission of clinical care, research, and education. Within medical education, virtual or distance learning combined with simulation became more common [1, 3]. Similarly, graduate medical and research education incorporated virtual didactic and telemedicine training [3–7]. Here, we describe the strategic pivot to the Virtual Summer Research Experience in Cancer (vSREC) from two traditional pipeline programs, aimed at immersing high school juniors, seniors, and early undergraduate students from underrepresented populations in biomedical science.

Providing early biomedical research opportunities has been shown to enhance future interest in biomedical careers [8]. Student-reported gains included disciplinary skills, research design, information or data analysis skills, information literacy, self-confidence, communication, and professional advancement [9–11]. Importantly, students from underrepresented backgrounds are particularly likely to benefit from early biomedical research experiences [12, 13]. These research experiences and the resulting sense of responsibility positively impact academic and career success after accounting for parental income and other factors that influence achievement [14]. In addition to focusing on diverse student trainees, teachers’ participation in research programs that include laboratory research and professional development can improve their students’ achievement in science [15].

Several National Cancer Institute (NCI)-designated cancer centers have instituted summer research programs (SRP) for high school and early undergraduate students underrepresented in biomedical research. Since 2003, the Indiana University Simon Comprehensive Cancer Center (IUSCCC) has provided summer research experiences to over 300 students, hereafter termed interns, from underrepresented populations, defined using the NIH definition of populations underrepresented in the extramural biomedical workforce (detailed in Table 1). In addition, in 2013, IUSCCC launched the Future Scientist Program (FSP), focusing on high school juniors in the Indianapolis Public School district, which contains a high percentage of disadvantaged students. The two-month-long programs not only provided first-hand research experience in cancer but also allowed students to develop long-term professional relationships with faculty mentors. Over 70% of interns have entered healthcare/science professions, and several have become physician-scientists, physicians, or biomedical scientists (unpublished data).

**Table 1:** NIH Definitions of students and underrepresented populations in science and from disadvantaged backgrounds

***Categories***	As defined by	Examples of groups
**Racial and ethnic groups**	National Science Foundation	Black, African-American, Hispanic, Latinos, American Indian, Alaskan Native, Native Hawaiian, Other Pacific Islanders
**Individuals with disabilities**	Americans with Disabilities Act	Visual, hearing, walking, lifting, or cognitive disabilities
**Disability as defined by at least two of the following subcategories** a) Homelessness b) Foster system c) Eligible for free or reduced lunch d) No parents with bachelor’s degree e) Eligible for Pell grants	McKinney-Vento Homeless Assistance Act Administration for Children and Families US Department of Agriculture ED.gov	
**Disadvantaged backgrounds** Grew up in rural and low income area	Health Resources and Services Administration Rural Health Grants Eligibility Analyzer or Center for Medicare and Medicaid Services-designated low income and health professional shortage area	

Previously, SRP and FSP had a similar structure: interns received a stipend to work on a research project in a faculty mentor’s laboratory (usually bench-based research) for 6–8 weeks, culminating in a poster and/or oral presentation. The laboratory experience was enriched by attendance at guest lectures on cancer biology and clinical cancer care, workshops on college/medical/graduate school applications and professional etiquette, and formal didactic training in research ethics, responsible conduct of research, and use of animals in research. Also, interns had social and celebratory events along with vertical mentoring opportunities with other trainees to teach how to network and navigate the university environment. IUSCCC also more recently initiated a 3–4-week high school teacher research program (TRP), placing teachers in research laboratories for hands-on experience. The aims of SRP and FSP were to expose interns to university-level cancer research through a full-time, paid summer program; to introduce concepts in cancer biology and medicine; to inspire interns to pursue further studies in science and/or medicine; and to build long-term relationships between mentors and interns.

**Fig. 1:**
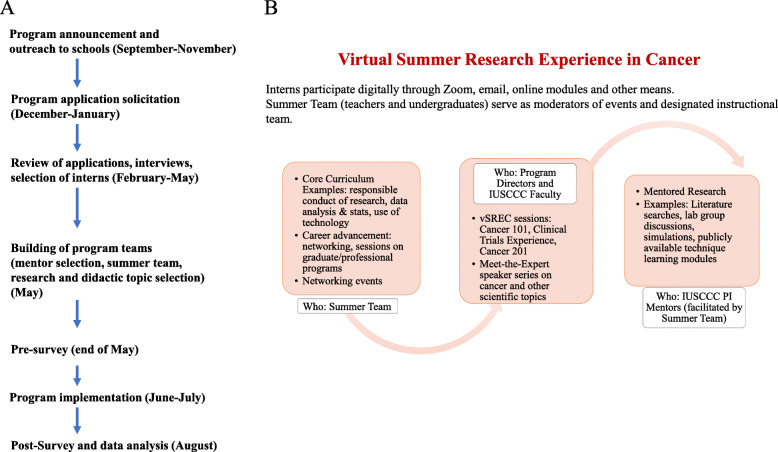
Schematic presentation of vSREC. **A** Timeline and workflow of vSREC. **B** vSREC core curriculum and participating teams and Faculty

Preparation to launch SRP, FSP, and TRP for the 2020 summer started in Fall 2019 (Fig. 1a), and application review, interviews, and candidate selection were almost complete just before the COVID-19 pandemic caused by the SARS-CoV2 novel coronavirus [16] forced a “hibernation” of research on our campus, pausing all but essential in-person research, as Indiana and much of the United States were placed under stay-at-home orders [17]. Since an in-person program became impossible, we opted to retool the curriculum as a virtual experience because of the importance of the programs in the lives of young interns, not just as a career-enhancing experience, but also as a full-time, stipend-based activity in a summer with few other options.

Here, we describe the modification of the traditional SRP and FSP pipeline programs into a virtual summer program, named Virtual Summer Research Experience in Cancer (vSREC), and the evaluation of this virtual program. The aims of vSREC were identical to FSP and SRP. Specific to vSREC, we sought to answer the following research questions: 1) What are interns’ perceptions of the impact of the vSREC program and their program mentor? 2) Do vSREC interns feel more able to understand and conduct research at the end of the program? and 3) What are mentors’ perceptions of the impact of the vSREC program on interns? This virtual pipeline program is unique in that it brought together a diverse group of high school and undergraduate students, high school teachers, IUSCCC leadership, and faculty mentors with a shared goal to provide a positive experience in early biomedical research.

## Methods

### Study participants

Students were selected for the program prior to the pandemic-related “hibernation”. Applicants submitted an online application including educational history, prior research experience, statement of interest, and a free-text description of how they met the eligibility criteria in Table 1. Trainees were selected for interview based on their applications, with final offers based on a holistic assessment by program faculty and staff of written and interview performance. All vSREC interns were invited to participate from May 2020 to July 2020. This study received approval from the Institutional Review Board at Indiana University (IRB protocol #1110007280) and written informed consent was obtained from all the participants. All procedures followed were in accordance with the ethical standards of the responsible committee on human experimentation and with the declaration of Helsinki.

### Design of the vSREC

Figures 1a and b provide a schematic timeline and overview of the vSREC; additional details on program design are in Additional file 1. The educational objectives of vSREC mirrored the objectives of the in-person programs of previous years as noted above: exposure to cancer research, introduction to cancer biology concepts, inspiration toward further science studies, and networking. For vSREC, we also added the objective of enhancing contemporary scientific literacy through education on virology and SARS-CoV2, as new knowledge in this area was moving incredibly rapidly in the summer of 2020, along with significant dissemination of misinformation [18]. Additionally, we aimed to provide hands-on training for our diverse trainees in dealing with microaggressions. This training was particularly timely during the Summer 2020 period of Black Lives Matter protests across the United States and recognition of racism, discrimination, and microaggressions in health care settings [19–22].

To meet all these objectives, vSREC utilized input and expertise in technology, teaching and evaluation, and cancer biology from a diverse group of individuals. Technology-adept local undergraduate students, who had completed rigorous science coursework, had a desire to pursue health- or science-related fields, had laboratory research experience, and/or had completed previous summer research programs on campus, provided hands-on and competent technology support, vertical mentoring, and campus navigation and networking advice. Local science teachers designed and delivered a 6-week core curriculum covering topics related to the research processes, scientific literacy, ethics, and grade-level resources for academic and career advancement.

**Fig. 2:**
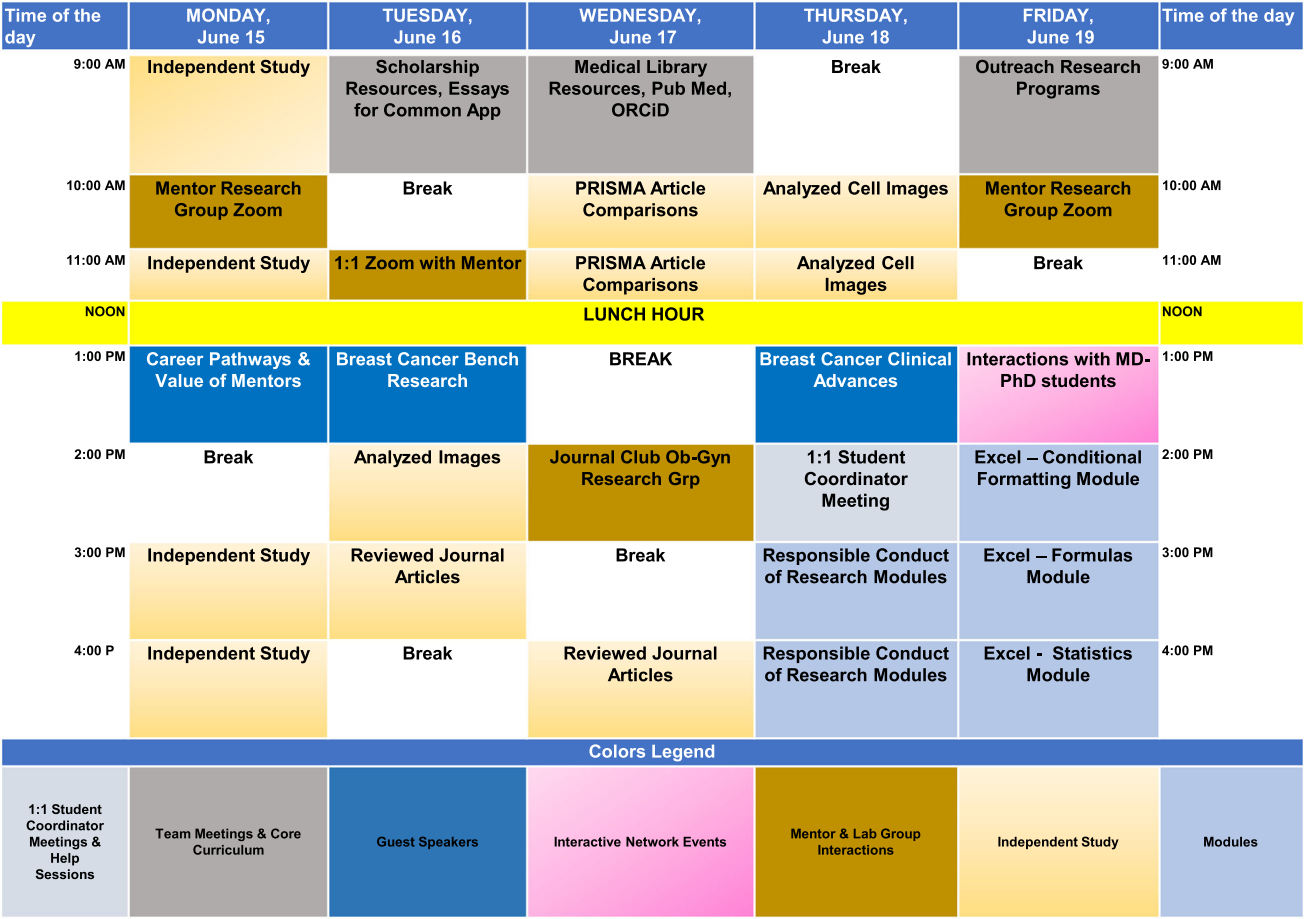
Representative vSREC weekly activity schedule. Activity of week 2 of the program for a specific intern is shown

IUSCCC faculty delivered engaging lectures in cancer biology, starting with fundamental cancer topics and moving through areas of specialty, while also modeling various career paths. Faculty also served as research project mentors along with their laboratory groups, providing virtual projects that could be done remotely. These included in silico analyses, literature reviews, and analyses of existing imaging or other datasets, plus virtual training in laboratory techniques. Finally, engaging networking events gave students the chance to interact with peers and others. Further details of these components are provided in Additional file 1. An example intern’s weekly program schedule and activities are depicted in Fig. 2, and Additional file 2 details all curriculum events, the daily checkout questions, and the extensive list of questions interns posed during the closing Cancer 201 lecture. This multifaceted approach to a virtual program allowed us to apply principles of online learning, including frequent contact between mentor and learner, clear organization, rapid feedback, diversity of presentation types and styles, a supportive learning environment, and appropriate training with online platforms for both students and mentors [23].

### Evaluation methods

The evaluation was conducted using a multi-method design where both quantitative and qualitative data were collected in surveys from interns and mentors. Survey instruments were created locally to evaluate the interns’ perceptions of the vSREC educational and research experience, their mentors, and the skills learned from the virtual experience using Likert-style and free response questions. Furthermore, we created an 11-item Research Preparation Scale to assess interns’ perceptions of their understanding of the research process and whether this improved as a result of the program. The Research Preparation Scale was created for use across all Indiana University summer research programs and contains items to evaluate and compare general aspects of research preparedness at the beginning and end of programs. The scale contains three domains: 1) Understanding of research process (6 items), 2) Research application (3 items), and 3) Applicable skills (2 items). In addition, a post-survey of mentors was created to collect their perceptions of the virtual program and its impact on their interns. Surveys were collected and managed using REDCap electronic data capture tools. REDCap (Research Electronic Data Capture) is a secure, web-based application designed to support data collection for research [24]. Copies of all surveys, none of which are under license, are provided in Additional file 3. At the beginning of the vSREC, interns were invited to complete a pre-survey that collected demographic information and the Research Preparation Scale that evaluated the degree to which interns felt prepared and able to conduct research. Further, using free responses, interns were asked what they hoped to learn from the program and to discuss any concerns about the virtual research experience.

Interns completed a post-survey at the end of the program that included the same 11-item Research Preparation Scale as well as items evaluating the overall research and educational experience and evaluation of the research mentor. Free responses collected data about what interns learned from the research experience, future career goals, and their overall perception of the virtual research experience.

Demographic information and responses to the post-survey are reported as descriptive statistics. A Cronbach’s α was performed on the Research Preparation Scale and domains to assess the reliability of the instrument. A Wilcoxon Signed Rank Test was used to compare interns’ responses on the Research Preparation Scale domains. Statistical analysis was done with SPSS v.27 (IBM Corp, Armonk NY) and significance was set at *p* ≤ 0.05.

Free response data from pre- and post-surveys were analyzed using the Framework Method of thematic analysis [25]. Data analysis using the Framework Method began with familiarization of the data by recording initial impressions. This was followed by the next stage, open coding, where all potentially relevant excerpts were marked. Codes were then aggregated into categories to create an analytical framework that was applied to the remaining responses. Finally, categories were reviewed and compared to results of the survey to illustrate and provide depth to the quantitative findings.

## Results

### Participant demographics

Intern demographics are detailed in Table 2 and included six Caucasian, 14 African American, one Asian, and one mixed-race interns.

**Table 2:** Intern demographics. *n* = 22 interns total

Characteristic	n (%)
Gender	
Male	4 (18%)
Female	18 (82%)
Educational level completed	
High school junior	8 (36%)
High school senior	5 (23%)
College freshman	9 (41%)
Race	
Caucasian	6 (27%)
African-American	14 (64%)
Asian	1 (5%)
Multi-Racial	1 (5%)
Ethnicity	
Hispanic/Latinx	3 (14%)
Non-Hispanic/Latinx	19 (86%)

### Pre-program concerns

All 22 interns in the vSREC completed the pre-survey. In a free-text response, eight interns expressed concerns about having a different research experience due to the virtual format. Specifically, they worried about not getting hands-on experience and having difficulty working with their mentors at a distance. Table 3 presents the results of the thematic analysis, including categories, codes, and representative quotes.

**Table 3:** Results of thematic analysis including codes and representative quotes

Categories	Codes	Representative Quote
Incoming Concerns	Lack of hands on experience	“I am a hands-on learner, so I am not sure how well I will understand new material if it is all just listening to others virtually.”
	Difficulty connecting with mentor	“My concern is not being able to connect with my mentor as well this summer because she will be busy and we won’t see each other every day like the in-person program”
Experience with vSREC Mentors	Created supportive environment	“My mentor was always available for questions via email or if, necessary, Zoom… Dr. --- was also very supportive by taking the time out of her schedule to meet with me 1:1 to discuss my progress.”
	Career advisors	“She supported me beyond the summer program. She helped guide me for future research in college.”
	Providing feedback	“During the Zoom call he always gave constructive feedback and told us how proud he was of us.”
Enjoyable aspects of vSREC	Guest speakers	“The most enjoyable aspects for me was listening to guest speakers and learning the range of paths in the field of science and research”
	Learning digital skills	“I learned how to use Zoom efficiently which will be important for the coming school year. I liked the professional tips we learned, like having a virtual background and having a profile picture or staying muted in meetings but being on camera.”
Improving the vSREC Experience	Match peers in labs	“Working with my mentor was nice but I would have liked being able to connect with another student in my lab. The others were just older than me and not really doing the same kind of summer experience.”
	Reducing screen time	“It was difficulty having back-to-back meetings and sitting in the chair all day.”
	Increased interactions and networking	“I suggest having more opportunities for the interns to network with one another.”

### Program outcomes: interns

**Fig. 3:**
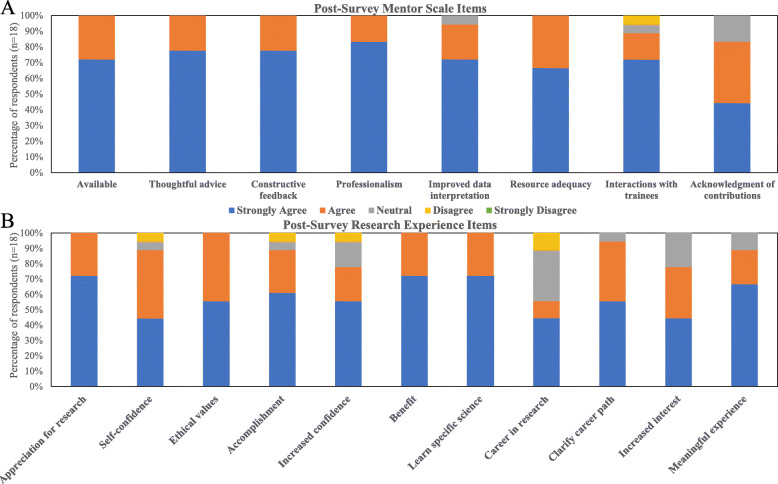
Evaluation of mentors and programs by vSREC interns. **A** Evaluation of mentors by interns. **B** The impact of vSREC on interns; results of post-program survey of *n* = 18 interns

A total of 18 interns completed the post-survey (12 in the SRP and six in the FSP, 82% response rate). All interns agreed or strongly agreed that their mentors were available to answer questions, provide advice, feedback, and resources to complete their research project (Fig. 3a). Interns further described how mentors supported them in the summer research experience by making themselves available to answer questions, provide feedback, and offer advising beyond the program. Others described how their mentors were able to create a supportive learning environment. One intern stated:

*He created such a friendly and informative atmosphere. My mentor was very engaging and friendly during all our interactions, which definitely made me comfortable and content with my internship. In addition, he was able to explain very complex ideas in such a wonderful way! He started with the basics, then added fun anecdotes, until we could finally fully understand the more complex material. This helped to keep my interest level extremely high throughout all our interactions as well as during my independent study. His passion definitely rubbed off on me!*

Each intern rated the vSREC experience as good or excellent. Each intern also self-reported gaining a greater appreciation for research, learning ethical conduct of research, and studying a topic in depth. Fifty-five percent agreed or strongly agreed that they wanted to pursue a career in research (Fig. 3b). Interns found expert speakers to be the most enjoyable aspects of the vSREC, followed by networking events. They appreciated the speakers sharing their research experiences and the pathways they took in their careers to reach their goals. One intern described:

*The most enjoyable aspect of the virtual summer research experience was being able to hear all these different guest speakers who had such different paths that they followed to achieve their goal. It was overwhelming and very encouraging to continue chasing my dream after hearing all the bad experiences and setbacks that they experienced yet still managed to overcome.*

Interns also discussed the digital skills they learned during the virtual program and how they planned to use these in the future. Interns discussed how many of the skills in Zoom, Google Drive, and Canvas would assist them in college, particularly in online courses.

While the interns overwhelmingly enjoyed the virtual program, many discussed their challenges and recommendations for future virtual programs. Nearly half of respondents expressed a desire to have a hands-on research experience and described the difficulty in sitting in front of their computer for several hours a day. Interns recommended having more activities that were interactive to build rapport and engagement among interns and mentoring staff and to try to match interns in a lab with others at their level of education.

**Fig. 4:**
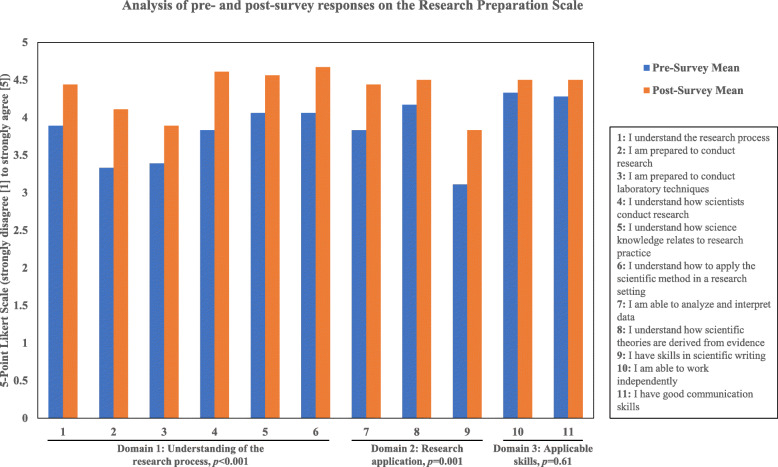
Comparative analysis of pre- and post-program survey results of interns’ pre-vSREC expectations and experience of vSREC, grouped by domain. *n* = 18 interns

The Research Preparation Scale was included on both the pre- and post-surveys to evaluate interns’ perceptions of their understanding of and ability to conduct research after completing the virtual program (Table 4). Interns reported significantly higher scores in the Understanding of the research process (*p* < 0.001) and Research application (*p* = 0.001) domains at the end of vSREC. There was no difference across the Applicable skills domain (*p* = 0.138) (Fig. 4). Cronbach’s α revealed the scale had acceptable to good reliability across the instrument (α = 0.80) and within each domain (1 = 0.78, 2 = 0.72; 3 = 0.61) (Table 4).

**Table 4:** Domains of the research preparation scale, reliability, and pre- and post-survey means

Domain	1. Understanding of the research process	2. Research application	3. Applicable skills
**Survey Questions**	1. I understand the research process. 2. I am prepared to conduct research. 3. I am prepared to conduct laboratory techniques. 4. I understand how scientists conduct research. 5. I understand how science knowledge relates to research practice. 6. I understand how to apply the scientific method in a research setting.	7. I am able to analyze and interpret data 8. I understand how scientific theories are derived from evidence 9. I have skills in scientific writing	10. I am able to work independently 11. I have good communication skills
**Cronbach’s α**	0.78	0.72	0.61
**Pre-survey mean ± SD**	3.76 ± 0.601	3.70 ± 0.523	4.31 ± 0.572
**Post-survey mean ± SD**	4.41 ± 0.392	4.24 ± 0.469	4.50 ± 0.707
***p*-value**	*< 0.001*	*0.001*	0.138

### Program outcomes as assessed by mentors

**Fig. 5:**
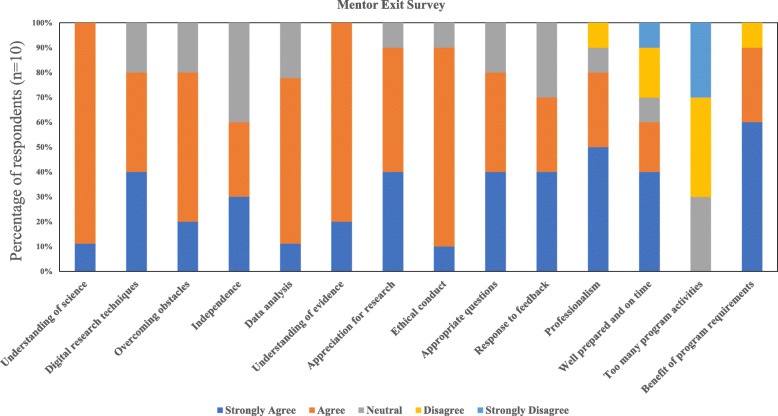
Evaluation of vSREC interns by mentors; results of post-program survey of *n* = 10 mentors

At the end of the program, mentors were also sent a REDCap survey to assess their opinion of their interns’ progress. Of 10 respondents (out of 17 mentors), all agreed or strongly agreed (on a 5-point Likert scale) that “my intern gained understanding of how scientists work on real problems,” while 80% agreed or strongly agreed that “my intern learned digital research techniques” and “asked appropriate questions” (Fig. 5). No mentors felt that interns spent too much time on other program activities, and 90% agreed/strongly agreed that program requirements enhanced the experience. However, 30% of mentors felt that interns were not on time or well prepared for Zoom meetings and commented anecdotally about varying levels of engagement and lack of clarity of expectations for both mentors and interns.

## Discussion

### Program outcomes: summary

As the COVID-19 pandemic unfolded in the USA, leading to lab hibernation in late March 2020, IUSCCC program leaders prioritized creation of these alternatives to hands-on research experiences. Due to stress associated with COVID-19, the abrupt closure of schools, and the resulting loss of social networks, the leadership of vSREC had tepid expectations for the program and expected student attrition. However, all but one student selected for the program based on an interview before hibernation readily accepted the offer to participate in vSREC. Moreover, each intern finished the program. The self-reported student and mentor outcomes strongly suggest a high degree of satisfaction with the program.

### Pre-program perception and post-program assessment about knowledge and skill improvements

Although it was not the primary intention of the program, trainee and mentor surveys provided us a glimpse of knowledge and skill improvements in a virtual mode (Figs. 4 and 5). While interns expressed reservations about connecting with mentors in pre-program surveys, the majority were satisfied with their interactions with mentors and how their interactions with mentors helped them to improve their cancer knowledge. Overall, there was a consensus that the virtual program was effective in enhancing cancer knowledge. However, expectedly, the virtual format was not considered ideal to improve hands-on skill development, including communication skills, a fact acknowleged by both interns and mentors. And engagement through long days of virtual sessions was a challenge: 30% of mentors felt that interns were not on time or were not as prepared as they would have liked. Considering the prevailing COVID-19 situation at the time of the program implementation and uncertainty regarding school reopening and potential career/educational opportunities in the near future, we feel that the overall commitment of interns to the program was remarkable and above the expectations of program leaders. However, there is an opportunity for further improvements, which are described below and summarized in Table 5.

**Table 5:** Lessons learned from the vSREC program

Mentors and Interns	Program Implementation
Mentors provide a link to research projects and model scientific career paths	Integrating high school teachers strengthens the program by providing curriculum and personal interactions with trainees
Mentors provide lectures for disciplinary relevance	The undergraduate “Summer Team” facilitators provide near-peer mentoring and technology support
Generation of rewarding virtual research projects is an ongoing challenge	Good internet connectivity is a requirement for successful participation
Interns value the professional development training but could benefit from professional communication/scheduling content	Clear training on integrating multiple technology platforms is necessary to avoid confusion
Clear expectations for mentor-trainee interactions are needed	Frequent check-ins minimize trainee disengagement

### Lessons learned: mentors and interns

The research mentors provided a crucial link to research projects, models of cancer research career paths, and discipline-specific lecture topics, ensuring a cancer research focus was maintained. In the future, we envision additional innovative virtual projects in the areas of bioinformatics, image analysis, literature searches, and other in silico lab topics.

For interns, future plans might focus more on professional communication. This training would include how to create a calendar-based schedule, how to schedule meetings on a mentor’s calendar, and professional etiquette for timeliness. Clearer expectations related to intern-mentor interactions during the course of the program, such as having interns present at laboratory meetings, may further improve program experience, mentor satisfaction, and outcomes.

### Lessons learned: program implementation

Active participation of high school teachers was key to the success of this program, as they applied their teaching and student-teacher interaction skills to keep interns engaged during the entire program. They also designed the curriculum shared by all interns, providing a common point of reference for all program participants. In the future, it will be valuable to draw on teacher expertise to design tests of student knowledge pre- and post-program, to ensure that self-reported learning achievements are supported by unbiased metrics.

Recruiting teachers for summer programs may pose a challenge in the future. Currently, teachers are seeing fewer opportunities for professional development within their schools because more time is being taken up to troubleshoot and prepare for the health and safety of the students within the virtual and in-person teaching platforms. This, in turn, creates fewer opportunities to enlist other strong teachers to assist with the summer programs. A further challenge is that teachers are working longer hours to develop virtual and in-person lessons to accommodate the hybrid calendars created by most schools. This gives them less personal time to participate in professional development activities such as the Teacher Research Program. In the future, similar programs will need to consider innovative ways to recruit and retain strong teachers to help facilitate these high school pipeline programs.

The undergraduate students of the Summer Team were an invaluable part of the program, providing near-peer mentoring and technological support for online tools. The availability of computing devices and a good internet connection is a limiting factor for any virtual program. In an ideal program, tablets with cellular data connections would be made available to interns who need them. Although using multiple learning management system (LMS) platforms allowed for more comprehensive functionality than opting for a single standalone platform, this multi-platform use caused confusion for the interns, as they often struggled to remember the purpose of each platform. However, the benefits of this multi-platform method included access to the different native tools within each platform. No one platform provides all of the features needed to run a wholly virtual program. Still, training on integrating external platforms such as G Suite and Zoom into a central LMS system such as Canvas can help reduce some of the confusion that interns faced during the vSREC experience. Also, more comprehensive pre-program IT training for all interns by a member of the institution’s educational IT support team could help better prepare students for the upcoming program.

### Limitations

This study was limited by the evaluation instruments. The surveys were primarily self-reported perceptions of the program rather than outcome measures. Further, while the locally created Research Preparation Scale has adequate internal consistency, it has not been evaluated for construct validity and other psychometric properties. In addition, the study population was small.

### Future directions

Future goals include performing a confirmatory factor analysis on the Research Preparation Scale and publishing the results. Further, we plan to evaluate the long-term outcomes of the program through an alumni survey. We also hope to assess knowledge gained in the virtual curriculum compared with face-to-face cohorts; such a study design was not possible within the constraints of the rapid transition to a virtual program in 2020.

The IUSCCC SRP program typically gets > 200 applications for 15–17 slots. Thus, many students with interest in cancer research do not get the opportunity to participate. Further, IUSCCC summer programs do not provide a residential option, so many students from rural communities may be disadvantaged from participating. The virtual programs, however, offer the opportunity to engage students beyond geographic proximity to National Cancer Institute-designated cancer centers, particularly for those cancer centers that have entire states as their catchment area. This opportunity can be explored in future.

Virtual research experiences, such as those examples described here, also offer a chance for meaningful engagement in cancer research to interns previously hindered by limited mobility. Engaging students with limited mobility (i.e., long-term wheel-chair bound or temporary injury limited mobility students) in the laboratory is challenging [26]. While the rehabilitation field has used adaptive sports as therapy [27], the adaptation of equipment and research facilities has been less swift. A virtual program could offer an appealing option for such students. As work from home and telehealth becomes more accepted, we envision innovative opportunities to increase.

## Conclusions

We transitioned a summer research program in cancer to a virtual experience by drawing on the expertise of researchers, high school teachers and college students. We provided cancer-related and professional development didactic sessions, virtual research activities, and networking. Trainees reported high satisfaction with their virtual experience. Such a program is not only useful in future situations that require virtual learning, but also could be implemented on a routine basis to provide summer research opportunities to students from rural school districts, non-research-intensive universities, or universities not affiliated with a cancer center or medical school. Thus, a program such as this, developed in response to COVID-19, can potentially change the depth and breadth of cancer education. These impactful programs allow cancer centers to engage with communities. Although we hope that IUSCCC will be in a position to offer a hands-on laboratory experience in future years, our virtual framework provides an appealing and effective alternative if needed.

## Supplementary Information

**Additional file 1:** Detailed Program Design.

**Additional file 2:** Tables presenting vSREC final program, daily checkout survey, and Cancer 201 questions.

**Additional file 3:** Surveys used in this study.

## Data Availability

The datasets used and/or analyzed during the current study are available from the corresponding author on reasonable request.

## Abbreviations

COVID-19: Coronavirus disease 2019
FSP: Future Scientist Program
IUSCCC: Indiana University Simon Comprehensive Cancer Center
LMS: Learning management system
NCI: National Cancer Institute
REDCap: Research Electronic Data Capture
SRP: Summer Research Program
TRP: Teacher Research Program
vSREC: Virtual Summer Research Experience in Cancer
