# Supplementary material for: Building a virtual summer research experience in cancer for high school and early undergraduate students: lessons from the COVID-19 pandemic
Source: BMC Med Educ. 2021 Aug 9;21:422. doi: 10.1186/s12909-021-02861-y (PMC8350276; doi:10.1186/s12909-021-02861-y)
Supplement: Supplementary file 3 — Additional file 3: Surveys used in this study. [file 12909_2021_2861_MOESM3_ESM.pdf]

CTSI STEM Programs Pre Research Experience Questionnaire

Please complete the survey about your interest in the Project STEM research experience below. We greatly value your honest responses.

All responses will remain confidential and will be used for program assessment and research purposes only.

An Evaluation of Student and Alumni Perceptions of Summer Research Experiences for High School and Undergraduate Students

We are doing a research study. A research study is a special way to learn about something. We are doing this research to learn more about the potential for positive impact of summer research experiences on the students who participate in our programs. We would like to ask you to be in this research study.

You are being asked to be in this research study because we wish to learn more about your summer research experience and the thoughts you have about it.

This survey will take 15-20 minutes to complete and will ask you questions about your future academic plans, about the summer research experience, and about how to improve the summer research experience.

Official LAST NAME. Please provide your LAST NAME as it appears on your social security card, or other similar official documents. If your last name is more than one word, please include it here with with caps, or no caps the way it appears on your documents -example: de la Vega.

____

Official FIRST & MIDDLE NAME/S. Please provide your FIRST NAME - and if you have - MIDDLE NAME - as they appear on your social security card, or other similar official documents. Some people have more than 1 middle name so in this field please enter all your OFFICIAL names that are NOT your last name.

____

Email address

____

Please select one or more options from the list below to describe your gender identity:

- ☐ Woman
- ☐ Man
- ☐ Transgender
- ☐ genderqueer, gender fluid
- ☐ Another identity not listed
- ☐ Prefer not to disclose

For another identity not listed, please specify:

____

How do you self-identify? Select all that apply.

☐ American Indian/Alaska Native
☐ Asian
☐ Black or African American
☐ Latino, or of Spanish origin
☐ Middle Eastern
☐ Native Hawaiian/Other Pacific Islander
☐ White
☐ Other
☐ Prefer not to disclose

For other, please specify:

____

What is the highest level of education completed by your Mother.

◯ Less than high school
◯ High school or equivalent (GED)
◯ Technical or occupational certificate
◯ Associate degree (2-year college degree)
◯ Some college coursework completed
◯ Bachelor's degree (4-year college degree, BA, BS) ◯ Master's degree (MA, MS, MBA, etc.) ◯ Professional degree (MD, DDS, JD etc.) ◯ Doctorate degree (PhD, EdD, etc.)
◯ I do not know

What is the highest level of education completed by your Father.

◯ Less than high school
◯ High school or equivalent (GED)
◯ Technical or occupational certificate
◯ Associate degree (2-year college degree)
◯ Some college coursework completed
◯ Bachelor's degree (4-year college degree, BA, BS) ◯ Master's degree (MA, MS, MBA, etc.) ◯ Professional degree (MD, DDS, JD etc.) ◯ Doctorate degree (PhD, EdD, etc.)
◯ I do not know

Regardless if you applied to STEM or SEED, please review the Federal Poverty Guidelines for Household Income that is used to determine Stipend Eligibility for both the SEED & STEM programs and select YES if you would qualify and NO if you would not. You may review the scale according to the number of people in your household and focus on the 200% column at this link:
200 Percent Federal Poverty Guidelines Link

◯ Yes ◯ No ◯ I do not know

In which program will you participate this summer?

◯ vSRP
◯ vFSP
◯ STEM
◯ Physics
◯ ICU-SRP

Which option best describes your educational level?

- ◯ I finished high school grade 10, will start grade 11
- ◯ I finished high school grade 11, will start grade 12
- ◯ I finished high school grade 12, will start undergrad year 1
- ◯ I finished undergrad year 1, will start undergrad year 2
- ◯ I finished undergrad year 2, will start undergrad year 3
- ◯ I finished undergrad year 3, will start undergrad year 4
- ◯ I finished undergrad year 4, will start undergrad year 5
- ◯ Other

For Other, please specify

____

Thoughts on Continuing Education

I am interested in pursuing a 4-year college degree.

◯ Strongly Disagree ◯ Disagree ◯ Neither Agree nor Disagree ◯ Agree ◯ Strongly Agree

I plan to declare a science-based major in college.

◯ Strongly Disagree ◯ Disagree ◯ Neither Agree nor Disagree ◯ Agree ◯ Strongly Agree

I want to do this experience to enhance my resume.

◯ Strongly Disagree ◯ Disagree ◯ Neither Agree nor Disagree ◯ Agree ◯ Strongly Agree

I want to do this research experience to gain experience working in a research laboratory.

◯ Strongly Disagree ◯ Disagree ◯ Neither Agree nor Disagree ◯ Agree ◯ Strongly Agree

Prior to having participated in a Summer Research Experience, rate the degree to which you agree with the following statements:

	Strongly Disagree	Disagree	Neither Agree nor Disagree	Agree	Strongly Agree
I understand the research process.	◯	◯	◯	◯	◯
I am prepared to conduct research.	◯	◯	◯	◯	◯
I am prepared to conduct laboratory techniques.	◯	◯	◯	◯	◯
I am able to work independently.	◯	◯	◯	◯	◯
I am able to analyze and interpret data.	◯	◯	◯	◯	◯

I understand how scientific theories are derived from evidence.	○	○	○	○	○
I understand how scientists conduct research.	○	○	○	○	○
I have skills in scientific writing.	○	○	○	○	○
I have good communication skills.	○	○	○	○	○
I understand how science knowledge relates to research practice.	○	○	○	○	○
I understand how to apply the scientific method in a research setting.	○	○	○	○	○

Please answer the following questions as they relate to the Virtual Summer Research Experience.

What is your level of proficiency with using e-learning platforms (e.g., Canvas, Blackboard, Brightspace, Oncourse, Moodle)

○ I have never used an e-learning platform ○ Not at all proficient ○ Somewhat proficient
○ Very proficient

Please describe any concerns you have beginning this virtual research experience.

Additional Thoughts, Comments, Feedback?

What do you hope to learn from this research experience?

How do you think this research experience will impact your future career choice?

Please share any additional thoughts, comments, or concerns about the summer research experience.

Summer Research Post-Experience Questionnaire

Please complete the survey about your summer research experience. We greatly value your honest responses.

All responses will remain confidential and will be used for program assessment and research purposes only.

An Evaluation of Student and Alumni Perceptions of Summer Research Experiences for High School and Undergraduate Students

We are doing a research study. A research study is a special way to learn about something. We are doing this research to learn more about the potential for positive impact of summer research experiences on the students who participate in our programs. We would like to ask you to be in this research study.

You are being asked to be in this research study because we wish to learn more about your summer research experience and the thoughts you have about it.

This survey will take 30-45 minutes to complete and will ask you questions about your future academic plans, about your experiences in the summer research program, and about how to improve the experience.

Name (First Last)

____

Email address

____

Please select your program:

- ◯ vSRP
- ◯ vFSP
- ◯ STEM
- ◯ Physics
- ◯ ICU-SRP

Which of the following best describes your educational level:

- ◯ I finished college year 4
- ◯ I finished college year 3
- ◯ I finished college year 2
- ◯ I finished college year 1
- ◯ I finished high school and will enter college this fall
- ◯ I will enter grade 12 this fall
- ◯ I will enter grade 11 this fall

As a consequence of participating in this summer research experience, rate the degree to which you agree with the following statements:

I am interested in pursuing a 4-year college degree.

- ◯ Strongly disagree
- ◯ Disagree
- ◯ Neither agree nor disagree
- ◯ Agree
- ◯ Strongly Agree

I plan to declare a science-based major in college.

- ◯ Strongly disagree
- ◯ Disagree
- ◯ Neither agree nor disagree
- ◯ Agree
- ◯ Strongly Agree

Since having participated in this Summer Research Experience, rate the degree to which you agree with the following statements:

	Strongly disagree	Disagree	Neither agree nor disagree	Agree	Strongly Agree
I understand the research process.	◯	◯	◯	◯	◯
I am prepared to conduct research.	◯	◯	◯	◯	◯
I am prepared to conduct laboratory techniques.	◯	◯	◯	◯	◯
I am able to work independently.	◯	◯	◯	◯	◯
I am able to analyze and interpret data.	◯	◯	◯	◯	◯
I understand how scientific theories are derived from evidence.	◯	◯	◯	◯	◯
I understand how scientists conduct research.	◯	◯	◯	◯	◯
I have skills in scientific writing.	◯	◯	◯	◯	◯
I have good communication skills.	◯	◯	◯	◯	◯
I understand how science knowledge relates to research practice.	◯	◯	◯	◯	◯
I understand how to apply the scientific method in a research setting.	◯	◯	◯	◯	◯

Based on your Summer Research Experience, rate the degree to which you agree with the following statements:

	Strongly disagree	Disagree	Neither agree nor disagree	Agree	Strongly Agree
I have a greater appreciation for scientific research.	○	○	○	○	○
My participation has improved my self-confidence.	○	○	○	○	○
I have learned values of ethical conduct in research.	○	○	○	○	○
I feel like I have accomplished something important this summer.	○	○	○	○	○
I have increased confidence my ability to conduct research.	○	○	○	○	○
I have benefitted (other than financially) from having this summer experience.	○	○	○	○	○
This experience allowed me to learn about a specific scientific topic in depth.	○	○	○	○	○
I want to pursue a career in research.	○	○	○	○	○
My research experience has helped clarify my career path.	○	○	○	○	○
I have an increased interest for the field in which I performed research.	○	○	○	○	○

Rate the degree to which you agree with the following statements about your research mentor:

	Strongly disagree	Disagree	Neither agree nor disagree	Agree	Strongly Agree
My lab mentoring staff was available to answer questions.	○	○	○	○	○
My lab mentoring staff provided thoughtful advice.	○	○	○	○	○
My lab mentoring staff offered constructive feedback.	○	○	○	○	○
The head mentor was professional toward students.	○	○	○	○	○
My mentoring staff helped develop my capacity for data interpretation.	○	○	○	○	○

Resources were adequate to complete my research project.	○	○	○	○	○
My head mentor promoted interactions with other trainees/students.	○	○	○	○	○
My head mentor acknowledged my contributions as a trainee.	○	○	○	○	○

What things made your mentor or mentoring staff supportive?

Overall how would you rate the experience you had with your mentor and mentoring staff?

○ Excellent ○ Good ○ Fair ○ Poor

Based on your virtual research experience, rate the degree to which you agree with the following statements:

	Strongly disagree	Disagree	Neither agree nor disagree	Agree	Strongly Agree
The virtual program offered a meaningful learning experience.	○	○	○	○	○
I enjoyed the sessions with guest speakers.	○	○	○	○	○
The training modules were beneficial.	○	○	○	○	○

What is the likelihood you would be involved with the program if it were offered again virtually?

○ Very likely ○ Somewhat likely ○ Not at all likely

Have you been involved with a Summer Research Experience prior to this virtual research experience?

○ Yes ○ No

Given your experience being involved with Summer Research Experience in-person, how to you think the virtual program can be improved if it has to be done again?

What in the program was lost by moving to a virtual research experience? What was gained?

Please provide additional thoughts, comments, and feedback in the questions below:

Please describe any barriers you experienced while participating in the virtual program.

Please describe any online research skills you learned from your Summer Research Experience being offered virtually and how you plan to use those skills in the future.

Please describe any other digital skills you learned from your Summer Research Experience being offered virtually and how you plan to use those skills in the future.

What were the most enjoyable aspects of the virtual Summer Research Experience?

What were the least enjoyable of the virtual Summer Research Experience?

What suggestions or recommendations do you have for the virtual Summer Research Experience?

2020 vSRP + vFSP Mentor Exit Survey

Please complete the survey about your most recent mentoring experience with your vSRP and/or vFSP student. We greatly value your honest responses.

All responses will remain confidential and will be used for program assessment and research purposes only.

NOTE: If you had one student in FSP and also one in SRP please complete the survey for each. Thank you

---

Mentor Name (First Last)

____

PI Name (First Last) (if different from above)

____

Mentee Name (First Last)

____

For which program did you serve as a mentor?

○ vFSP
○ vSRP

Research Experience

Please answer the following questions to the best of your knowledge, referring to your most recent IUSCCC vFSP or vSRP student mentee:

In my opinion, my mentee gained understanding of how scientists work on real problems.

○ Strongly Agree ○ Agree ○ Neutral ○ Disagree ○ Strongly Disagree

---

My mentee learned digital research techniques.

○ Strongly Agree ○ Agree ○ Neutral ○ Disagree ○ Strongly Disagree

---

My mentee learned that overcoming obstacles is an integral part of research.

○ Strongly Agree ○ Agree ○ Neutral ○ Disagree ○ Strongly Disagree

---

My mentee learned to work independently.

○ Strongly Agree ○ Agree ○ Neutral ○ Disagree ○ Strongly Disagree

---

My mentee enhanced his/her ability to analyze and interpret data.

○ Strongly Agree ○ Agree ○ Neutral ○ Disagree ○ Strongly Disagree

My mentee better understands that scientific theories and conclusions, to some extent, are based on evidence.

◯ Strongly Agree ◯ Agree ◯ Neutral ◯ Disagree ◯ Strongly Disagree

My mentee now has an increased appreciation for scientific research.

◯ Strongly Agree ◯ Agree ◯ Neutral ◯ Disagree ◯ Strongly Disagree

My mentee learned values of ethical conduct in research.

◯ Strongly Agree ◯ Agree ◯ Neutral ◯ Disagree ◯ Strongly Disagree

Evaluation of Research Mentee and Educational Experience

My mentee asked appropriate questions.

◯ Strongly Agree ◯ Agree ◯ Neutral ◯ Disagree ◯ Strongly Disagree

My mentee responded well to advice and constructive feedback.

◯ Strongly Agree ◯ Agree ◯ Neutral ◯ Disagree ◯ Strongly Disagree

My mentee showed respect and consideration and was professional.

◯ Strongly Agree ◯ Agree ◯ Neutral ◯ Disagree ◯ Strongly Disagree

My mentee was on time and well prepared for all Zoom meetings

◯ Strongly Agree ◯ Agree ◯ Neutral ◯ Disagree ◯ Strongly Disagree

My mentee spent too much time participating in other program activities (guest speakers etc.)

◯ Strongly Agree ◯ Agree ◯ Neutral ◯ Disagree ◯ Strongly Disagree

Program requirements (mandatory presentations, CITI modules, etc.) enhanced my mentee's summer experience

◯ Strongly Agree ◯ Agree ◯ Neutral ◯ Disagree ◯ Strongly Disagree

My mentee was a good match for me in terms of interest in the project.

◯ Strongly Agree ◯ Agree ◯ Neutral ◯ Disagree ◯ Strongly Disagree

The $1,000 bench fee was adequate to support the mentee's research.

◯ Strongly Agree ◯ Agree ◯ Neutral ◯ Disagree ◯ Strongly Disagree

Overall how would you rate the experience you had with your mentee?

◯ Excellent ◯ Good ◯ Fair ◯ Poor

What factors make a mentee successful?

Additional Thoughts, Comments, and Feedback

What were the best aspects of vFSP?

What were the best aspects of vFSP?

What were the worst aspects of vSRP?

What were the worst aspects of vFSP?

What suggestions or recommendations do you have for vSRP?

What suggestions or recommendations do you have for vFSP?

If you have any additional comments or feedback that you would like to share regarding the summer research program, please type them below.
